# Development of a Measure of Postpartum PTSD: The City Birth Trauma Scale

**DOI:** 10.3389/fpsyt.2018.00409

**Published:** 2018-09-18

**Authors:** Susan Ayers, Daniel B. Wright, Alexandra Thornton

**Affiliations:** ^1^Centre for Maternal and Child Health Research, City, University of London, London, United Kingdom; ^2^Alder Graduate School of Education, Redwood City, CA, United States

**Keywords:** birth, PTSD, questionnaire, postpartum, measure, trauma, scale

## Abstract

Post-traumatic stress disorder (PTSD) affects 4% of women after birth yet there are very few questionnaire measures of postpartum PTSD that have been validated in this population. In addition, none of the available questionnaires assess postpartum PTSD in accordance with criteria specified in the latest edition of the Diagnostic and Statistical Manual [DSM-5, (1)]. The City Birth Trauma Scale is a 29-item questionnaire developed to measure birth-related PTSD according to DSM-5 criteria of: stressor criteria (A), symptoms of re-experiencing (B), avoidance (C), negative cognitions and mood (D), and hyperarousal (E), as well as duration of symptoms (F), significant distress or impairment (E), and exclusion criteria or other causes (H). Two additional items from DSM-IV were also included on the basis of evidence suggesting they might be important in this population. The first was criterion A2 that women responded to events during birth with intense fear, helplessness or horror. The second was symptoms of emotional numbing. Items were first reviewed by researchers (*n* = 9) and postpartum women (*n* = 8) and revised accordingly. The questionnaire was then completed by 950 women recruited online. Results showed the City Birth Trauma Scale had excellent reliability (Cronbach's α = 0.92) and is easy to understand (Flesch reading score 64.17). Exploratory factor analysis found two factors which together accounted for 56% of the variance: (i) Birth-related symptoms (40.8% variance) and (ii) General symptoms (15.5% variance). PTSD symptoms were highly associated with distress, impaired functioning, and women reporting they wanted treatment (*r* = 0.50–0.61). Removing DSM-IV A2 criteria only increased births classified as traumatic by 2%. Adding the item on emotional numbing did not change the psychometric properties of the scale. These items were therefore removed. The City Birth Trauma Scale has good psychometric properties and the two symptom clusters identified are consistent with previous research on symptoms of postpartum PTSD. This scale therefore provides a promising measure of PTSD following childbirth that can be used in research and clinical practice. Future research should examine the scale's predictive validity using clinical interviews.

## Introduction

Reviews and meta-analyses show postpartum PTSD affects 3–4% of all women after birth and 15 to 19% of women in high risk situations such as those who have pregnancy complications or preterm birth (2, 3) and up to 39% of women whose babies die (4). The main risk factors for postpartum PTSD are depression in pregnancy, fear of childbirth, negative birth experiences, complications of pregnancy or birth, lack of support and dissociation during birth (5). Postpartum PTSD is also highly comorbid with depression (5). However, despite this evidence, PTSD still remains largely unrecognized in maternity services and, unlike depression, is not routinely screened for. Affected women are therefore rarely identified and treated for PTSD.

One of the possible barriers to this is the lack of validated questionnaires that measure postpartum PTSD. Research in this area has typically adapted questionnaires developed for use in other groups, such as military veterans, which may not be applicable to women after birth. Perinatal research comparing general measures of PTSD with specific measures of postpartum PTSD shows that, although they are highly correlated, agreement between these scales on identification of diagnostic cases of PTSD is low (6). Measurement is therefore critical in identifying cases of postpartum PTSD. It is also important to examine which symptoms are most predictive of distress, impaired functioning, and the need for treatment in postpartum women.

A key issue to consider in perinatal research is which diagnostic criteria are appropriate when looking at PTSD in pregnant or postpartum women. The criteria for PTSD in the American Psychiatric Association Diagnostic and Statistical Manual changed quite substantially between the fourth edition (DSM-IV, (7)) and the fifth edition (DSM-5 (1)). For an event to be classed as a traumatic stressor DSM-IV diagnostic criteria specified that the event needs to involve “actual or threatened death or serious injury, or a threat to the physical integrity of self or others” (criterion A1) and the person responded to this with intense fear, helplessness, or horror (criterion A2). Complications during childbirth can lead to actual or perceived threat to the life of women and/or their baby which can result in intense negative emotions therein fulfilling criteria A1 and A2 for a traumatic stressor. Three clusters of PTSD symptoms were specified: re-experiencing, avoidance and numbing, and hyperarousal symptoms.

In DSM-5 the diagnostic criteria were revised. Criterion A2 and emotional numbing were removed. Eight of the original 17 symptoms were edited, and a further three symptoms added to create a fourth cluster of symptoms of “negative cognitions and mood” (1). This was done on the basis of empirical evidence and clinical observations to incorporate the wide range of trauma symptoms people can present with (8). However, it has been criticized as being overly complex and resulting in diagnostic discordance. Glatzer-Levy and Bryant (9) calculated that there were 636,120 possible combinations of symptoms to meet the diagnosis of PTSD. Reviews of the factor structure of PTSD using DSM-IV and DSM-5 are also inconclusive, with most support for four or five factors in DSM-IV and six or seven in DSM-5 PTSD (10).

The implications of DSM-5 changes to PTSD criteria for measuring postpartum PTSD are not clear. There is some evidence that removing criterion A2 may result in up to twice as many births being classified as traumatic (11, 12). In an Australian study of 890 women, Boorman et al. (11) found that the proportion of births that met stressor criteria increased from 14.3 to 29.4% when A2 was removed. This may be because it is common for women to perceive events during birth as potentially injuring or threatening to their physical integrity but do not necessarily fear this. It might also be due to how stressor criteria are measured, and that questionnaires need to be worded so that women only fulfill A1 if they perceive a threat of severe injury or death.

Similarly, DSM-5 removed emotional numbing and replaced it with anhedonia (an inability to experience positive emotions). This has been criticized as ignoring the evidence that emotional numbing is one of the most predictive symptoms of chronicity and impairment (13). In mothers who have been exposed to trauma there is also evidence that emotional numbing is more predictive of parenting stress than other PTSD symptoms (14).

Another issue is that PTSD symptoms might be inflated in the postpartum period by normal postpartum factors, such as fatigue and increased vigilance toward the baby. For example, there is evidence in other populations that sleep disturbances are common features of PTSD (15) but also important in the onset of mental illness (16) and the persistence or maintenance of chronic PTSD (17). Thus, the sleep deprivation associated with caring for an infant could inflate, confound or contribute to PTSD at this time.

Similarly, motherhood and routine postpartum healthcare may make it hard for women to avoid reminders of birth, such as health clinics and the baby, which could lead to fewer symptoms of avoidance. Perinatal research has examined this in relation to symptoms of hyperarousal. Ayers et al. (18) compared hyperarousal symptoms in 1,049 women in the UK who had a traumatic birth or a non-traumatic birth and found that, although mean hyperarousal symptoms were significantly greater in women who had a traumatic birth, the diagnostic criteria of two or more hyperarousal symptoms (measured in the previous week) were also met by 50% of women who did not have a traumatic birth.

Two questionnaires have been developed specifically for PTSD in pregnancy or after birth. The first is the Traumatic Event Scale (19). This scale follows the diagnostic criteria from DSM-IV (7) and can be used in pregnancy to measure PTSD related to the upcoming birth, or after birth to measure PTSD as a response to birth events. The Traumatic Event Scale has been widely used in perinatal research and has good face validity, but has not been tested psychometrically or validated against clinical interviews. The second measure is the Perinatal PTSD Questionnaire, which was originally developed for use with high-risk mothers (20). It has since been modified to improve clinical utility (21). The modified version is a 14-item scale which does not ask about stressor criteria or cover all the diagnostic criteria. However, it does include items that measure avoidance, intrusions, arousal, and negative cognitions and mood. This scale therefore has a few items that measure each of the PTSD symptom clusters specified in DSM-5, but is not a diagnostic measure. Thus, there are no questionnaires that measure postpartum PTSD according to all DSM-5 diagnostic criteria.

The aim of this study was therefore to develop a questionnaire measure of PTSD specifically for postpartum women that measures PTSD according to DSM-5 criteria. As part of this we will examine the appropriateness of specific diagnostic criteria for postpartum women, such as whether it is better to retain A2 and emotional numbing, and whether the new symptom cluster of negative cognitions and mood is coherent in postpartum samples. This study will therefore provide a measure of postpartum PTSD according to current diagnostic criteria for use in research and clinical practice.

## Materials and methods

### Development of the city birth trauma scale

Initial questions were written to correspond directly with the DSM-5 criteria (criterion A, symptoms B-E, onset, duration, and distress) but be specific to childbirth. These initial questions were then reviewed by two groups to check face validity, content validity, and ease of comprehension. First, the questions were reviewed by a group of nine researchers with expertise in perinatal mental health research. These researchers ranged from doctoral to professorial level and were from psychology or mental health nursing. After that, questions were revised and then reviewed by a second group of eight women aged 21–34 who were members of the maternal health Research Advisory Group at the university and whose youngest children were aged between 13 months and 4 years. As a result of review by these groups, changes were made to the instructions of the questionnaire and wording of many items.

This development stage resulted in a questionnaire that comprised 31 questions: 29 questions which map onto DSM-5 diagnostic criteria; and 2 questions from DSM-IV criteria (one for criterion A2; and one on symptoms of emotional numbing). The question on A2 was included on the basis of evidence that it might be important in postpartum PTSD. The question on emotional numbing was included following review by the group of postpartum women who strongly recommended it be retained because of its relevance to them and potential impact on the mother-baby relationship.

The response scale for symptoms asks for frequency of symptoms over the last week and is scored on a scale ranging from 0 (“not at all”) to 3 (“5 or more times”). A higher score indicates greater severity of symptoms of PTSD. Diagnostic criterion A items were scored on a yes/no scale. Distress, disability and potential physical causes were rated as yes/no with a third response category of “maybe” for women who were not sure. The “maybe” category was added after review by postpartum women (as above). A copy of the City Birth Trauma Scale is available as online Supplementary Material.

### Validation of the city birth trauma scale

#### Procedure

Psychometric properties of the questionnaire were examined by asking postpartum women to complete the questionnaire online. Ethical approval was obtained from the University Research Ethics Committee. Women were eligible for the study if they had had a baby within the previous 12 months and were aged 16 years or over. The questionnaire was put online using Qualtrics software (22) for 3 weeks in August 2015. Women were recruited through social media (e.g., Twitter, Facebook) and webpages (e.g., City, University of London). Participation was voluntary and anonymous unless women chose to give their email address to receive information about the results of the research.

Prior to participating, women were given information about the study and had to tick a box to confirm they were 16 years or over and consented to take part in the study. If they did not tick this box they could not continue to participate in the survey. Women who provided consent then completed the City Birth Trauma Scale, demographic information, obstetric information, and perceived need for treatment. *Demographic and obstetric information* comprised basic demographic (age, ethnicity, relationship status) and obstetric details (parity, time since birth, type of birth i.e., normal vaginal, assisted vaginal, emergency cesarean or elective cesarean). *Need for treatment* was included to examine which symptoms were most predictive of need for treatment. Women were asked whether they had had professional help or treatment (“have you had professional help or treatment for these symptoms?”) or wanted professional help or treatment for their symptoms (“do you want professional help or treatment for these symptoms?”). The response scale was yes/no/maybe. Women who said “yes” or “maybe” to either of these items were categorized as needing treatment.

At the end of the survey women were thanked for their help and given information about websites and helplines they could contact if they had been affected by any of the symptoms in the questionnaire. Finally, if women wanted to receive a copy of the results they could enter their email address which was kept separately from the data file so that responses were anonymized.

#### Sample

1,018 postpartum women participated and completed some or all of the questionnaire. Of these, 68 women were not eligible because their baby was born more than 12 months previously. The final sample for analyses was therefore 950 women, of whom 117 had some missing questions on the City Birth Trauma Scale. The number of women in each analysis therefore varies and is specified when reporting results. Women ranged in age from 18 to 46 years (mean = 32.25 years, SD = 4.62. Time since birth ranged from 0 to 12 months (mean = 5.61 months, SD = 3.47). Other sample characteristics are shown in Table 1.

**Table 1:** Sample characteristics.

		**% (*n*)**
Ethnicity	White	93.3 (758)
	Black/African/Caribbean	0.2 (2)
	Asian	2.6 (21)
	Mixed or multiple ethnic groups	2.5 (20)
	Other	1.3 (11)
Relationship status	Married/Living with partner	96.8 (793)
	Partner but not co-habiting	1.0 (8)
	Single/Separated/Divorced	2.2 (18)
Type of birth	Emergency C/S	16.2 (154)
	Elective C/S	7.4 (70)
	Assisted vaginal	16.2 (167)
	Normal vaginal	58.8 (559)
Parity	1	52.6 (429)
	2	36.6 (298)
	3	8.8 (72)
	4+	1.9 (16)

*Missing data means the total N ranges from 799 to 950*.

#### Validity and reliability

Some aspects of *Validity* were examined. Ease of comprehension was examined using the Flesch readability scale and years of formal education required using the Gunning Fog index. Construct validity was examined using exploratory factor analysis to see whether the four symptom subscales specified by the DSM-5 were observed in this sample; and whether including the additional item on emotional numbing affected the coherence of subscales. *Reliability* was examined using two indicators of internal consistency: First Cronbach's alpha coefficient, which is a measure of the interrelatedness between a set of questions designed to measure an overall construct. A minimum value of 0.7 is considered acceptable for a new scale (23). And second the alpha values when individual questions were removed.

Analyses were conducted using R 3.2.3 (24), pROC (25) and SPSS 20.

## Results

### Screening of items

Initial data screening was conducted to examine whether any questions were not performing well. First, the range of each question was examined to ensure there were no restricted questions where participants did not use the full range of the scale. This confirmed all questions used the full range. Secondly, the distributions of the questions were examined through inspection of skewness values and histograms. PTSD scales are frequently skewed when used in normal populations and this was expected in this sample of women. Eighteen of the 20 symptom questions in the City Birth Trauma Scale were positively skewed, therefore it was not appropriate to remove questions on this basis. Finally, questions were screened and noted if they were too highly correlated with other questions *r* > 0.90 (0 questions) or did not significantly correlate with other questions (0 questions). One question had low correlations with the other PTSD symptom questions (“Not able to remember details of the birth” range 0.09–0.31). The other correlations were higher.

### PTSD symptoms

Descriptive statistics for frequency of responses to each item of the questionnaire are provided in online Supplementary Materials. The total PTSD symptom score was calculated by adding together the scores for each of the items. Total PTSD symptoms had a possible range of 0–60. In the current sample, total PTSD symptoms ranged from 0 to 56 with a mean score of 11.70 (SD 11.06). The majority of women in the sample (90.6%) reported one or more symptoms. Scores for percentiles were: 25th = 3, 50th = 9, 75th = 18.

Responses to stressor criteria are shown in Table 2. It can be seen that 14.4% of women thought they or their baby would die, and 15.6% thought they would be seriously injured. Substantially more women reported a response of intense negative emotions (A2) at 37.4%. Stressor criteria A for a traumatic birth were fulfilled by 18.3% of the sample using DSM-IV criteria and 20.3% using DSM-5 criteria. Using DSM-5 stressor criteria on the City Birth Trauma Scale therefore increased the number of women identified as fulfilling stressor criteria by 2%. Seventy-two women (7.8%, CI 6.08–9.52%) fulfilled all DSM-5 diagnostic criteria for PTSD. Six of these women indicated their symptoms might be due to medication, alcohol, drugs or physical illness. Excluding these women meant 7.1% of the sample met diagnostic criteria for PTSD (CI 5.45–8.75%).

**Table 2:** PTSD and stressor criteria.

	**No % (*n*)**	**Yes % (*n*)**
Did you believe you or your baby would be seriously injured?	84.4 (798)	15.6 (148)
Did you believe you or your baby would die?	85.6 (810)	14.4 (136)
Did you feel any intense negative emotions (e.g., fear, helplessness, horror)?	62.6 (592)	37.4 (354)
Fulfill DSM-IV criterion A	81.7 (773)	18.3 (173)
Fulfill DSM-5 criterion A	79.7 (754)	20.3 (192)
DSM-5 PTSD	92.2 (857)	7.8 (72)
DSM-5 PTSD removing women who meet possible exclusion criteria	92.9 (863)	7.1 (66)

Responses to questions about onset of symptoms, duration, distress and impairment are shown in Table 3. This shows the majority of women with symptoms reported onset as within the first 6 months after birth (75.0%); and that these symptoms had lasted 3 months or more (50.8%). However, a proportion of women reported their symptoms started before the birth (18.1%), suggesting they had pre-existing PTSD or related symptoms.

**Table 3:** Onset and impact of PTSD in women who reported symptoms.

**Item**	**Response scale**	**% (*n*)**
Onset of symptoms	Before the birth	18.1 (102)
	First 6 months after birth	75.0 (422)
	More than 6 months after birth	6.9 (39)
Duration of symptoms	Less than 1 month	20.6 (115)
	1–3 months	28.6 (160)
	More than 3 months	50.8 (284)
Do these symptoms cause you a lot of distress?	Yes	26.3 (151)
	Maybe	34.1 (196)
	No	39.7 (228)
Do they prevent you from doing things you usually do?	Yes	19.8 (113)
	Maybe	25.4 (144)
	No	54.9 (313)
Have you had professional help or treatment for these symptoms?	Yes	21.6 (122)
	Maybe	1.4 (8)
	No	77.0 (434)
Do you want professional help or treatment for these symptoms?	Yes	16.3 (89)
	Maybe	22.5 (123)
	No	61.2 (334)
Could these symptoms be due to medication, alcohol, drugs, or physical illness?	Yes/Maybe	7.8 (44)

Severity of symptoms was not associated with time since birth (*r* = −0.03). Just over a quarter of women said their symptoms caused them a lot of distress (26.3%) and one fifth said the symptoms prevented them from doing things (19.8%). Treatment had been obtained by 21.6% of women and a further 16.3% of women said they wanted treatment.

### Reliability

The total scale and subscales had good internal consistency with Cronbach's alphas above the acceptable level of 0.7 (α = 0.92 for total scale, 0.88 for intrusions, 0.83 for negative cognitions and mood, 0.86 for hyperarousal). Removing items from the total scale, intrusions or hyperarousal subscales did not improve the Cronbach's alpha. However, removing the item “Not able to remember details of the birth” slightly improved the internal consistency of the subscale for negative cognitions and mood (α changed from 0.83 to 0.86). It was not possible to examine Cronbach's alpha for the avoidance subscale because it only has two items, which were highly correlated (*r* = 0.71).

### Validity

The reading level of the questionnaire was established as easy to read at a level that would easily be understood by 13–15 year olds (Flesch Reading Ease score = 64.17). The number of years of formal education required to easily read the scale (Gunning Fog index) was 6.71.

The 20 items measuring symptoms from DSM-5 were entered into an exploratory factor analysis using maximum likelihood extraction and varimax rotation. This factor analysis excluded the item on emotional numbing. Statistical checks confirmed the sample was adequate for factor analysis (Kaiser-Meyer-Olkin measure = 0.94) and correlations between questions were suitably large (Bartlett's test of sphericity, χ^2^(190) = 10,283.1, *p* < 0.001). An orthogonal rotation (varimax) was used on the item loadings and the results are shown in Table 4. Two factors were identified which accounted for 56.3% of the variance. The first factor was “Birth-related symptoms” (eigenvalue 8.16, 40.8% of variance) and the second factor was “General symptoms” (eigenvalue 3.09, 15.5% of variance). The factors were moderately correlated with each other (*r* = 0.45).

**Table 4:** Exploratory factor analysis of the City Birth Trauma Scale.

	**Item loading by symptom cluster^a^**	**Factor analysis of all symptoms^b^ (varimax rotation)**	
		**Birth-related symptoms**	**General symptoms**
**INTRUSIONS**			
Recurrent unwanted memories of the birth (or parts of the birth) that you can't control.	0.80	0.78	
Bad dreams or nightmares about the birth (or related to the birth).	0.65	0.63	
Flashbacks to the birth and/or reliving the experience.	0.63	0.61	
Getting upset when reminded of the birth.	0.88	0.86	
Feeling tense or anxious when reminded of the birth.	0.90	0.88	
**AVOIDANCE**			
Trying to avoid thinking about the birth.		0.80	
Trying to avoid things that remind me of the birth (e.g., people, places, TV programs).		0.67	
**NEGATIVE MOOD AND COGNITIONS**			
Not able to remember details of the birth.	0.20		
Feeling negative about myself or thinking something awful will happen.	0.66		0.59
Blaming myself or others for what happened during the birth.	0.46	0.69	
Feeling strong negative emotions about the birth (e.g., fear, anger, shame).	0.51	0.79	
Lost interest in activities that were important to me.	0.83		0.76
Feeling detached from other people.	0.87		0.79
Not able to feel positive emotions (e.g., happy, excited).	0.81		0.78
**HYPERAROUSAL**			
Feeling irritable or aggressive.	0.76		0.65
Feeling self-destructive or acting recklessly.	0.55		0.59
Feeling tense and on edge.	0.86		0.73
Feeling jumpy or easily startled.	0.65		0.62
Problems concentrating.	0.71		0.64
Not sleeping well because of things that are not due to the baby's sleep pattern.	0.50		0.50
Feeling emotionally numb^c^			0.63^c^
**Total variance explained**		40.8%	15.5%

a This analysis looked at each symptom cluster separately and whether items within each cluster loaded onto it. It was not possible to do this analysis for avoidance because there are only two symptoms.

b *Loadings equal to or greater than 0.4 which did not cross load are shown. ^c^This DSM-IV item was added in a separate factor analysis and loaded on the factor for General symptoms. The loadings and factors for this second factor analysis were similar to those shown here*.

Associations between PTSD symptoms and women's distress, impairment and need for treatment are shown in Table 5. It can be seen that all subscales are significantly correlated with these outcomes, particularly distress and need for treatment (*r* = 0.30–0.62). Impaired functioning was more highly associated with general symptoms such as negative cognitions and mood, and hyperarousal (*r* = 0.51–0.65) than with birth-related symptoms such as intrusions or avoidance (*r* = 0.11–0.17).

**Table 5:** Correlations between PTSD symptoms, need for treatment, distress and disability.

	**Need for treatment**	**Distress**	**Impairment**
Intrusions	0.33	0.37	0.16
Avoidance	0.30	0.39	0.21
Negative cognitions and mood	0.54	0.59	0.53
Hyperarousal	0.51	0.53	0.54
Total PTSD symptoms	0.56	0.61	0.50
Factor 1: birth-related symptoms	0.38	0.40	0.20
Factor 2: general symptoms	0.57	0.61	0.63

*Pearsons r, all significant at p < 0.001*.

### Emotional numbing

Examination of whether the item on feeling emotionally numb added to the reliability and validity of City Birth Trauma Scale suggest it does not detract from the scale's psychometric properties. Including this item did not affect Cronbach's alpha for the scale (α = 0.92). Similarly, when the factor analysis was repeated with the emotional numbing item included it did not alter the pattern of results. The factor structure and loadings remained the same and the emotional numbing item loaded on the factor for “General symptoms” (item loading 0.63; factor eigenvalue 3.19; 15.2% of variance). Correlations between emotional numbing and measures of distress and impairment and need for treatment were moderate (*r* = 0.46 and 0.41, respectively) and weaker than correlations between these variables and the DSM-5 item on anhedonia which replaced it (inability to feel positive emotions, *r* = 0.61 and 0.50, respectively).

## Discussion

This paper reports the development of the City Birth Trauma Scale to measure postpartum PTSD according to DSM-5 criteria, as well as examining whether two items from DSM-IV may be particularly relevant to this population (A2 and emotional numbing) and need to be retained. The results suggest the City Birth Trauma Scale has good reliability and indication of reasonable validity. Total PTSD symptoms were highly associated with distress, impaired functioning and need for treatment.

This scale therefore provides a promising tool for assessing postpartum PTSD. However, two issues need to be considered in relation to the construct and measurement of postpartum PTSD. The first is whether items on DSM-IV criteria A2 and emotional numbing add significantly to the measure. The second is whether symptoms are best conceptualized in terms of the four symptom clusters proposed in DSM-5 or the two symptom clusters found here. These issues are discussed in turn.

### Should DSM-IV criteria A2 and emotional numbing be retained for postpartum PTSD?

Previous research on postpartum PTSD found that removing criterion A2 increased the number of women who fulfill stressor criteria by up to two times (11, 12, 26). However, this large increase was not observed in this study. Using DSM-5 A1 criteria only increased the proportion of women classified as having had a traumatic birth by 2% from 18.3 to 20.3%. In fact, many more women endorsed the A2 item of feeling intense negative emotions during birth (37.4%) than the A1 items of thinking they or their baby would be seriously injured or die (14.4 and 15.6%, respectively). It is possible the wording of DSM-5 A1 items in the City Birth Trauma Scale therefore removes the problem of over-inflation of cases by wording it as threat of serious injury or death. The 20.3% of women fulfilling DSM-5 A1 criteria on the City Birth Trauma Scale is similar to that found in other UK samples. For example, Ayers et al. (26) found 19.7% of women from community samples fulfilled stressor criteria using DSM-IV criteria. Thus removing A2 from the scale is unlikely to artificially inflate the prevalence of postpartum PTSD.

Similarly, the item on emotional numbing was included because of the relevance to postpartum women and evidence that it is most predictive of parenting stress in postpartum women (14). However, results showed that including this item did not alter or detract from the psychometric properties of the City Birth Trauma Scale. We therefore recommend that A2 and emotional numbing are removed from the City Birth Trauma Scale. However, researchers examining the impact of postpartum PTSD on parenting stress and the mother-baby relationship might like to include emotional numbing as a separate variable.

### Symptom structure of postpartum PTSD

Analysis of the construct of postpartum PTSD found it was best explained by two factors of birth-related symptoms and general symptoms. This is consistent with previous studies of the factor structure of postpartum PTSD which found the same two symptom clusters (6, 26, 27). The symptom subscales of intrusions and avoidance loaded onto the birth-related factor, and the hyperarousal subscale loaded onto the general symptom factor. However, a review of research in other populations found six or seven factors best explained PTSD as measured by DSM-5 (10). Also, in our sample the subscale of negative cognitions and mood which was added in DSM-5 was not coherent and loaded across both factors. One item from this subscale did not load on either factor (“not able to remember details of the birth”). Removing this item increased the internal reliability of the subscale of negative cognitions and mood. This suggests this item might not be as relevant to postpartum women—possibly because memory for birth is unlike other traumatic stressors in that birth experiences are subject to more attention, discussion and repetition, making it more likely that details are remembered. It is possible this item is therefore more relevant to other trauma populations.

### Limitations

A few limitations need to be considered before reaching conclusions. This sample was recruited online so is self-selected and potentially not representative of postpartum women. Demographic characteristics indicate the sample had a higher proportion of white women compared to the UK population (93% compared to 87%) although other characteristics are broadly representative. It is important that future research therefore examine the psychometric properties of City Birth Trauma Scale in ethnic minority populations.

Finally, this research was primarily focused on the development of a measure of postpartum PTSD and, as such, does not offer a validation because no tests of external validity or test-retest reliability were conducted. Future research is therefore needed to look at reliability over time and examine the predictive validity of the scale for identifying PTSD cases using clinical interviews to establish the validity of the scale as a diagnostic tool.

### Summary and conclusion

This study is the first to develop a specific measure of postpartum PTSD according to DSM-5 criteria. Results suggest the City Birth Trauma Scale has good reliability and there is some indication of validity. Consistent with previous research into postpartum PTSD it was best conceptualized as two symptom clusters of birth-related symptoms and general symptoms. Including the A2 criterion did not greatly alter the number of women fulfilling stressor criteria, so should be removed. Similarly, including the item on emotional numbing did not alter the scale's psychometric properties so can also be removed.

The City Birth Trauma Scale therefore provides a promising measure of birth-related PTSD for use in research and clinical practice. Future research should examine the psychometric properties of the City Birth Trauma Scale in different ethnic groups, and examine test-retest reliability and predictive validity compared to clinical interviews.

## Author contributions

SA developed and designed the questionnaire and research study, and drafted the manuscript. DW analyzed and interpreted the data and contributed to the manuscript. AT carried out the research and contributed to the manuscript.

### Conflict of interest statement

The authors declare that the research was conducted in the absence of any commercial or financial relationships that could be construed as a potential conflict of interest.
