# Supplementary material for: Development of a Measure of Postpartum PTSD: The City Birth Trauma Scale
Source: Front Psychiatry. 2018 Sep 18;9:409. doi: 10.3389/fpsyt.2018.00409 (PMC6153962; doi:10.3389/fpsyt.2018.00409)
Supplement: Supplementary file 1 [file Table_1.docx]

**Supplementary material: Frequency and mean score of responses to each symptom**

| **Intrusions** | Not at all  % (n) | Once  % (n) | 2-4 times  % (n) | 5 or more  % (n) | Mean (SD) |
| --- | --- | --- | --- | --- | --- |
| Recurrent unwanted memories of the birth (or parts of the birth) that you can’t control | 73.7(677) | 12.9 (118) | 9.3 (85) | 4.1 (38) | .44 (.83) |
| Bad dreams or nightmares about the birth (or related to the birth) | 90.8 (829) | 5.4 (49) | 2.2 (20) | 1.6 (15) | .15 (.52) |
| Flashbacks to the birth and/or reliving the experience | 64.0 (585) | 19.3 (176) | 12.0 (110) | 4.7 (43) | .57 (.88) |
| Getting upset when reminded of the birth | 72.9 (666) | 13.9 (127) | 8.0 (73) | 5.3 (48) | .46 (.85) |
| Feeling tense or anxious when reminded of the birth | 70.4 (643) | 15.8 (144) | 8.2 (75) | 5.7 (52) | .49 (.87) |
| **Avoidance** |  |  |  |  |  |
| Trying to avoid thinking about the birth | 77.6 (709) | 8.6 (79) | 6.2 (57) | 7.5 (69) | .44 (.91) |
| Trying to avoid things that remind me of the birth (e.g. people, places, TV programs) | 82.6 (757) | 6.3 (58) | 5.5 (50) | 5.6 (51) | .34 (.82) |
| **Negative Mood and Cognitions** |  |  |  |  |  |
| Not able to remember details of the birth | 75.9 (674) | 14.1 (125) | 6.8 (60) | 3.3 (29) | .37 (.75) |
| Feeling negative about myself or thinking something awful will happen | 67.9 (603) | 11.8 (105) | 12.5 (111) | 7.8 (69) | .60 (.98) |
| Blaming myself or others for what happened during the birth | 72.4 (643) | 13.4 (119) | 10.0 (89) | 4.2 (37) | .46 (.84) |
| Feeling strong negative emotions about the birth (e.g. fear, anger, shame) | 70.5 (626) | 14.0 (124) | 10.1 (90) | 5.4 (48) | .50 (.88) |
| Lost interest in activities that were important to me | 68.2 (606) | 13.6 (121) | 13.4 (119) | 4.7 (42) | .55 (.90) |
| Feeling detached from other people | 59.0 (523) | 16.9 (150) | 18.2 (161) | 6.0 (53) | .71 (.96) |
| Not able to feel positive emotions (e.g. happy, excited) | 68.2 (604) | 13.7 (121) | 14.2 (126) | 4.0 (35) | .54 (.88) |
| **Hyperarousal** |  |  |  |  |  |
| Feeling irritable or aggressive | 28.7 (247) | 21.5 (185) | 36.1 (311) | 13.7 (118) | 1.35 (1.04) |
| Feeling self-destructive or acting recklessly | 82.2 (707) | 9.8 (84) | 6.2 (53) | 1.9 (16) | .28 (.66) |
| Feeling tense and on edge | 34.0 (293) | 23.2 (200) | 30.1 (259) | 12.7 (109) | 1.21 (1.05) |
| Feeling jumpy or easily startled | 71.3 (614) | 11.8 (102) | 12.2 (105) | 4.6 (40) | .50 (.88) |
| Problems concentrating | 43.5 (374) | 17.8 (153) | 26.7 (230) | 12.0 (103) | 1.07 (1.09) |
| Not sleeping well because of things that are not due to the baby’s sleep pattern | 60.3 (519) | 16.5 (142) | 16.8 (145) | 6.4 (55) | .69 (.97) |
| **Dissociative symptoms** |  |  |  |  |  |
| Feeling detached or as if you are in a dream | 68.9 (593) | 14.8 (127) | 13.2 (114) | 3.1 (27) | .51 (.84) |
| Feeling things are distorted or not real | 82.3 (708) | 9.0 (77) | 6.3 (54) | 2.4 (21) | .29 (.69) |
| **Numbing** |  |  |  |  |  |
| Feeling emotionally numb (e.g. feeling sad but can’t cry, unable to have loving feelings) | 76.4 (658) | 10.9 (94) | 8.7 (75) | 3.9 (34) | .40 (.81) |
