# Supplementary material for: Development of a Measure of Postpartum PTSD: The City Birth Trauma Scale
Source: Front Psychiatry. 2018 Sep 18;9:409. doi: 10.3389/fpsyt.2018.00409 (PMC6153962; doi:10.3389/fpsyt.2018.00409)
Supplement: Supplementary file 2 [file Image_1.pdf]

CITY UNIVERSITY LONDON

Birth Trauma Scale

This questionnaire asks about your experience during the birth of your most recent baby. It asks about potential traumatic events during (or immediately after) the labour and birth, and whether you are experiencing symptoms that are reported by some women after birth. Please tick the responses closest to your experience.

What date was your baby born?_______________________________________

During the labour, birth and immediately afterwards:		
Did you believe you or your baby would be seriously injured?	Yes	No
Did you believe you or your baby would die?	Yes	No

The next questions ask about symptoms you might have experienced. Please indicate how often you have experienced the following symptoms in the last week:

Symptoms about the birth*	NOT AT ALL	ONCE	2 - 4 TIMES	5 OR MORE TIMES
Recurrent unwanted memories of the birth (or parts of the birth) that you can't control				
Bad dreams or nightmares about the birth (or related to the birth)				
Flashbacks to the birth and/or reliving the experience				
Getting upset when reminded of the birth				
Feeling tense or anxious when reminded of the birth				
Trying to avoid thinking about the birth				
Trying to avoid things that remind me of the birth (e.g. people, places, TV programs)				
Not able to remember details of the birth				
Blaming myself or others for what happened during the birth				
Feeling strong negative emotions about the birth (e.g. fear, anger, shame)				

* Although these questions refer to the birth, many women have symptoms about events that happened just before or after birth. If this is the case for you, and the events were related to pregnancy, birth or the baby then please answer for these events.

Symptoms that began or got worse since the birth	NOT AT ALL	ONCE	2 - 4 TIMES	5 OR MORE TIMES
Feeling negative about myself or thinking something awful will happen				
Lost interest in activities that were important to me				
Feeling detached from other people				
Not able to feel positive emotions (e.g. happy, excited)				
Feeling irritable or aggressive				
Feeling self-destructive or acting recklessly				
Feeling tense and on edge				
Feeling jumpy or easily startled				
Problems concentrating				
Not sleeping well because of things that are not due to the baby's sleep pattern				
Feeling detached or as if you are in a dream				
Feeling things are distorted or not real				

If you have any of these symptoms:

When did these symptoms start?	
Before the birth	
In the first 6 months after birth	
More than 6 months after birth	
Not applicable (I have no symptoms)	

How long have these symptoms lasted?	
Less than 1 month	
1 to 3 months	
3 months or more	
Not applicable (I have no symptoms)	

Do these symptoms cause you a lot of distress?	Yes	No	Sometimes
Do they prevent you doing things you usually do (e.g. socialising, daily activities)?	Yes	No	Sometimes
Could any of these symptoms be due to medication, alcohol, drugs, or physical illness?	Yes	No	Maybe

Thank you for completing this questionnaire
